# Predicting the virulence of future emerging zoonotic viruses

**DOI:** 10.1371/journal.pbio.3002286

**Published:** 2023-09-08

**Authors:** Samuel Alizon

**Affiliations:** Center for Interdisciplinary Research in Biology (CIRB), Collège de France, CNRS, INSERM, Université PSL, Paris, France

## Abstract

Would you rather kiss a platypus, a hedgehog or a llama? This study explores a new study in PLOS Biology which uses evolutionary epidemiology models to show that the life-history of the reservoir mammal hosts of zoonotic viruses might be the key to predicting their virulence in humans.

## Teaser

Would you rather kiss a platypus, a hedgehog, or a llama? According to a new study in this issue of *PLOS Biology*, the virulence of a zoonotic virus in humans depends on its reservoir host. Could physiology be the key to anticipating viral threats lethality?

Zoonotic viruses are recognised as a global threat. Although some of these like MERS-CoV or Ebola virus have shaped our imagination (and nightmares), should we generally expect them to be more virulent to humans than endemic viruses? Answering this question is difficult because zoonotic viruses are, almost by definition, poorly adapted to humans, and this maladaptation can either lead to low virulence (if the virus is rapidly cleared by the immune system) or huge virulence (by triggering immunopathological reactions such as cytokine storms).

So far, studies on the virulence of zoonoses have focused on transmission chains taking place right after the spillover. These showed, for example, that some infection life history traits, such as virulence, govern the probability of early extinction and rapid adaptation to humans [1]. More virulent strains can also be favoured in the early stages of a spillover when few hosts are immunised [2].

In a new study published in this issue of *PLoS Biology*, Brook and colleagues [3] tackle this long-lasting issue in an original way, using as a starting point the very reasonable assumption that zoonotic viruses should be adapted to their reservoir host. Their mathematical model and data analysis show that the physiological differences between reservoir and spillover hosts could be the key to understanding what makes a new virus virulent.

According to virulence evolution theory, the host life history matters. For example, in simple models, increased host life span and level of constitutive immunity are predicted to, respectively, favour less and more virulent strains. Mathematical models help to formalise these predictions, some of which have been validated experimentally [4].

Importantly, in this work, virulence is an intrinsic property of the virus. It has to be distinguished from the expressed virulence [5], or “lethality,” which also depends on the host and the environment. COVID-19 variants illustrated this mismatch recently: Even though its virulence was similar to that of the ancestral lineages, the Omicron BA.2 lineage was less lethal because human populations were different with widespread vaccination, postinfection immunity, and treatments [6]. The same is true for zoonoses: A virulent virus may be mild in its reservoir but lethal in humans because differences in physiologies lead to differences in expressed virulence.

**Fig 1:**
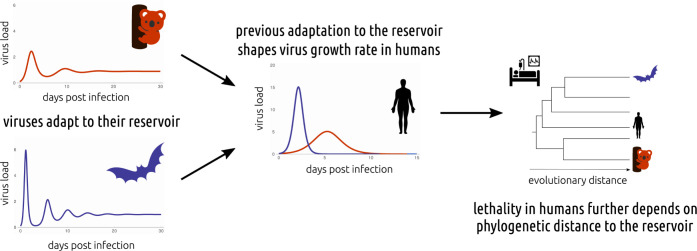
Zoonotic viruses are assumed to be adapted to their reservoir host. Brook and colleagues also assume that this adaptation shapes the growth rate of the virus and, hence, the within-host dynamics in humans. The lethality, or infection virulence incurred by the human host, is further shaped by the phylogenetic distance between the reservoir host and humans.

The literature on modelling within-host dynamics is dual, with deterministic models generating either chronic or acute infections. Brook and colleagues elegantly combine the two by assuming that zoonotic viruses cause chronic infections in their reservoir host and acute infections in their novel (human) host (Fig 1). Using an adaptive dynamics approach, they derive the “optimal” growth rate of the virus in its reservoir host, which they assume is retained, at least initially, following spillover to humans. Unfortunately, this growth rate depends on model parameters that can be difficult to estimate. Therefore, they use allometric theory to identify proxies for three key parameters (out of ten): the reservoir host mortality rate, its tolerance to the immunopathological reactions caused by the virus, and its constitutive immunity. Using publicly available databases, they can generate expected virus growth rates for reservoir species from 19 Mammal orders given estimates of their physiological parameters. From these growth rates, Brook and colleagues compute the expected virulence in humans while accounting for phylogenetic distance, following evidence that viruses from more distant hosts are less likely to be tolerated [7,8].

Overall, Brook and colleagues present us with the virulence that one might expect from a zoonotic virus coming from one of 19 potential reservoir hosts. Their predictions are consistent with data from 8 orders of mammals. This may appear as limited but it is the best one can do with the current data. In orders such as Chiroptera (bats), in which the authors infer a high tolerance to immunopathology and high levels of constitutive immunity, the expected (and observed) virulence is high. The expected virulence could be even higher in Monotrema (suggesting that you definitely should not kiss a platypus), but there currently is no data on emerging viruses from this order. Besides, the authors do not model the probability of emergence, and it is also likely that monotreme zoonoses are rare (or even nonexistent).

Overall, this work is a call for an improved understanding of the physiology of potential reservoir hosts, as well as virus ecology in general. This road map to detecting potentially virulent viruses in the wild also raises ethical concerns as to whether such a search should be undertaken (or at least how it should be implemented).

Other questions remain open. For instance, does this theory apply to non-Mammal reservoirs or to non-vertebrate hosts? Indeed, serial passage experiments already show that for arboviruses infecting humans, transmission rounds through arthropod vectors shape their evolution [9]. Furthermore, as noted by the authors, what about within-order differences?

The mathematical model also makes important assumptions that could be explored in the future. For example, the (intrinsic) growth rate of the virus is assumed to be constant in the reservoir and human hosts. Even the relative contribution of the virus replication to virulence through direct exploitation or immunopathology is assumed to be constant in both types of hosts. Overall, only the host tolerance and resistance parameters vary freely in the model. In terms of validation, could sampling be ill-balanced between all reservoir species? For example, less virulent infections could be more likely to be detected when originating from domesticated species.

An exciting possibility to validate the framework could be to apply it to emerging viruses in animal hosts. For example, the importance of the adaptation to the reservoir in shaping the virulence could be tested using a mouse system as the reference instead of humans. More generally, there is a need for additional laboratory studies such that key parameters could then be measured and not inferred via proxies.

Finally, the lethality of a zoonotic virus may be largely unrelated to the number of casualties it may cause since our ability to control an outbreak strongly depends on other factors, especially the basic reproduction number (*R*_0_) and the delay between contagiousness and symptoms [10]. For example, MERS or SARS are much more lethal than SARS-CoV-2 but had a more limited impact. Building on Brook and colleagues’ theory, we could envisage an extension of this work to study other traits than virulence to improve our detection of “virus X,” the virus likely to cause the next pandemic.
